# 

**Published:** 1874-04

**Authors:** 


### MAD RIVER VALLEY DENTAL SOCIETY.

The regular semi-annual meeting of the Mad River Valley Society, at the Phillips House, Dayton, Ohio, on Tuesday, April 7th, 1874, commencing at 10 o'clock A. M. sharp.

SUBJECTS FOR DISCUSSION.

FIRST.—What are the causes of wasting of the alveolus, and the consequent loss of the teeth, and what treatment is indicated?

SECOND.—Is there any difference between inflammation of the periosteum and that of any other tissue?

THIRD.—Is there any definite improvement in the operation of filling teeth within the last year?

FOURTH.—What improvements have been made in artificial dentures during the last year?

FIFTH.—What means will be most efficient for bringing the body of reputable dentists up to the present advance attainments?

SIXTH.—What discoveries and improvements have been made in dental practice in the past year?

SEVENTH.—What discoveries and improvements made within the last five years have stood the test of experience and are now in common use?

Executive Committee: Drs J. Taft, H. A. Smith and S. B. Tizzard.

Essayists: Drs S. B. Tizzard, R. H. Boal and A. J. Grosvenor.

G. W. Keely,

*President.*

J. A. Stipp,

*Rec. Secretary.*

### Editorial.

### THE DENTAL REGISTER,

### OF THE WEST.

### A Monthly Magazine, Devoted Entirely to the Interest of the DENTAL PROFESSION.

**Terms Reduced to $2.50 per Year. Single Nos. 25 Cents.**

**Edited by PROF. J. TAFT,**

**And Published by SPENCER & MOORE, of the Ohio Dental Depot**

The volume commences in January and closes in December.

The Register being issued monthly is always fresh, therefore indispensable to the practitioner who desires to keep posted up with the new inventions and improvements which are daily developed. Neither expense nor effort will be spared to give value and variety to its contents. Articles will be illustrated with well executed engravings whenever they will add to the interest or assist in explanation.

Its extensive circulation and frequency of issue makes it one of the BEST DENTAL ADVERTISING MEDIUMS in the United States.

All subscriptions must begin with January or July.

**RATES OF ADVERTISING:**

| | 1 mo. | 3 mo. | 6 mo. | 1 year. | Inserted pages | 1 year. | |
|---|---|---|---|---|---|---|---|
| 1 page ......... | $12 00 | $25 00 | $40 00 | $75 00 | must be plain | 1st page. | $50 00 |
| ½ page ......... | 8 00 | 15 00 | 25 00 | 40 00 | paper & black | 2d page. | 37 50 |
| ¼ page ......... | 5 00 | 10 00 | 15 00 | 25 00 | ink. | 3d page. | 33 33 |
| | | | | | | 4th page. | 25 00 |

Local or special notices, five lines or less, will be inserted for $3 each insertion; over five lines, 50 cents per line.

All advertising bills payable in advance, or at furthest, after the issue of the first number containing the advertisement. All transient advertisements to be paid for in advance.

☞ CONTRIBUTIONS SOLICITED.

Exclusively Editorial Communications should be addressed to

**DR. J. TAFT, EDITOR.**

The latest number of the Register may be ordered with or without other goods at any time, and all letters should be addressed to

**SPENCER & MOORE. PUBLISHERS,**

Cincinnati, Ohio.

